# Effect of Flushing the Endometrial Cavity With Follicular Fluid on Implantation Rates in Sub-Fertile Women Undergoing Invitro Fertilization: A Randomized Clinical trial 

**Published:** 2018-12

**Authors:** Kobra Hamdi, Mohammad Nouri, Sara Farzaneh, Mohammad Mirza-Aghdazadeh-Attari, Mohammad Naghavi-Behzad, Sahar Mohammadi

**Affiliations:** 1 Department of Gynecology and Obstetrics, Women’s Reproductive Health Research Center, Tabriz University of Medical Sciences, Tabriz, Iran; 2 Department of Community and Family Medicine, Medical Philosophy and History Research Center, Tabriz University of Medical Sciences, Tabriz, Iran; 3 Department of Community and Family Medicine, Students Research Committee, Tabriz University of Medical Sciences, Tabriz, Iran; 4 Department of Nuclear Medicine, University of Southern Denmark, Odense, Denmark; 5 Department of Emergency Medicine, Zanjan University of Medical Sciences, Zanjan, Iran

**Keywords:** Endometrial Cavity, Follicular Fluid, Invitro Fertilization, Implantation

## Abstract

**Objective:** To examine the effectiveness of intrauterine injection of follicular fluid in in vitro fertilization (IVF).

**Materials and methods:** A parallel randomized control clinical study was conducted on 110 patients attending Al–Zahra Educational-Medical Center of Tabriz University of Medical Sciences. Female candidates for IVF were categorized into intervention (n = 55) and control (n = 55) groups using Randlast software (version 1.2). Following an identical protocol of gonadotropin-releasing hormone (Gnrh) antagonist stimulating ovulation, in the intervention group a total of 2mL of follicular fluid was injected as intrauterine after the accomplishment of follicular puncture. Embryo transfer was carried out after 2-3 days. The rates of implantation, as well as chemical and clinical pregnancy were compared between the two groups.

**Results:** There were no significant differences in chemically proven pregnancy (19 in intervention group (34.5%) vs. 23 (41.8%) in control group p = 0.43), or in clinical pregnancy (30.9% vs. 38.2%, respectively p = 0.42), and in implantation rates (11.52 ± 2.57 % (range, 0-66.7) vs 18.79 ± 3.72 % (range, 0-100), respectively).

**Conclusion:** Injection of follicular fluid into the uterine cavity in candidates for IVF neither improves nor adversely affects the outcome of the therapy.

## Introduction

Infertility is defined as the lack of ability to conceive a child without the use of contraception in the time period of one year. One of the proposed methods to treat infertility is in vitro fertilization (IVF) (1). Although major breakthroughs were reported in assisted reproductive technologies during recent years, the success rates are still sub-optimal. A study done by Vasiliki A et al. showed that the cumulative success rate of IVF cycles were only around 50% (2). Also another study conducted by Luke et al. showed great potential in assisted reproductive technology (ART) methods in bridging the gap between infertile couples and fertile ones on the basis of cumulative pregnancy rate in 12 cycles, but also reported the dramatic distorting effect of shoddy treatment and environmental factors on results, and also demonstrated that implantation rates with seemingly healthy fetuses were low (3).

Success of ART methods are of great importance since they impose a great financial and emotional burden on the families undergoing treatment (4). It has been shown that the main culprit in the failure of ART procedures is the failure of implementation (50% to 75%) (1, 5), successful implantation depends on the quality of the embryo and the receptivity of the endometrium.

Receptiveness of the endometrium depends on estrogen, progesterone, a number of cytokines, growth factors, immunogenic factors (integrins and etc.) and other agents synchronizing the endometrium with the fetus.

Putting into perspective, one method to increase ART success rates is to tryto establish a hormonal balance and a receptive endometrium (5). 

Numerous endeavors are being considered to make the endometrium more receptive by the injection of hormones such as human chorionic gonadotropin (HCG) (6), cytokines and etc. to the endometrium, and pre-implantation endometrial scratching has also been proposed, but it has been with abysmal success (7). 

One of the key mediators suspected to have a positive effect on implantation is the natural follicular fluid. Follicular fluid is rich in growth factors such as stem cell growth factor, transforming growth factor, and also cytokines, which could have positive effects on implantation rates. Further notable is the immunomodulating effect of the follicular fluid, down regulating the expression of CD25, and inhibiting the synthesis of IL-1b and IL-2 (8). Moreover, it was suspected that follicular fluid could alter the secretory status of the endometrium (5). Regarding all of the findings above, it is possible that the therapeutic injection of follicular fluid could have a beneficial effect in increasing implantation rates, but only limited numbers of studies have been conducted to further investigate the role of the follicular fluid in increasing implantation rates and overall success of ART procedures. Therefore, this study was carried out to investigate the effect of endometrial flushing during ovary puncture, on implantation rate and IVF success rates.

## Materials and methods

**Figure 1:**
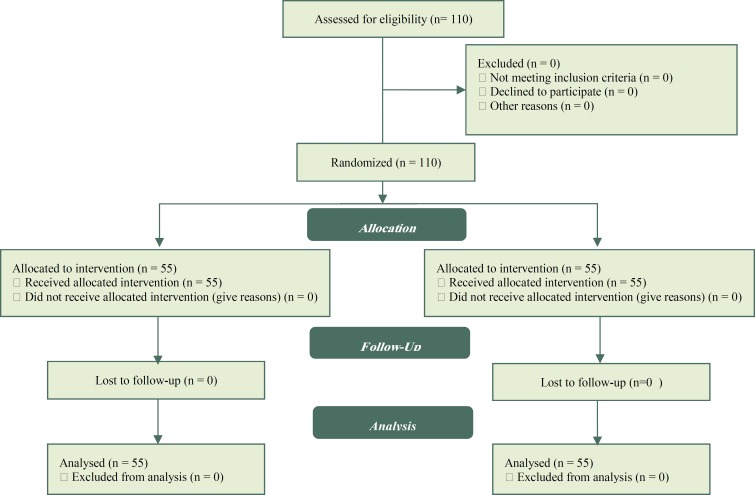
Consort diagram of the study

During the present clinical randomized controlled trial, which was conducted between June 2014 and June 2016 in Al-Zahra Educational-Medical Center of Tabriz University of Medical Sciences (Tabriz, Iran), 110 sub-fertile women were matched for age and other demographic futures, duration of infertility, not having a previous experience of IVF (first cycle IVF), and the levels of follicular stimulating hormone (FSH) in blood. The patients were randomly allocated to two groups including an intervention group, undergoing injection of follicular fluid to the endometrial cavity, and a second group as the control group. The two groups were compared in implantation and pregnancy rates. The consort diagram of the study is supplemented in figure 1. 

This study was registered at Iranian Registry of Clinical Trials (http://www.irct.ir) with the registration number of IRCT2015021713566N3 and the study protocol was approved by the Ethics Committee of Tabriz University of Medical Sciences number 1394.129, which was in compliance with the Helsinki Declaration. All patients signed informed written consent before inclusion in the study. Prior to every stage of the research project, patients were clearly informed of the procedures and had the ability of leaving the study at will. No harm resulting from the procedures was reported in the literature. 

Pregnancy rates were assigned as the primary results for a significance level of 0.05, power of 0.80, pregnancy rate of 40% and difference in clinical pregnancy rate of 10% between the two groups in favor of the intervention group (nonexistent). Then 110 women were randomized into two equal groups using Randlast software (version 1.2) and sealed opaque envelopes. Inclusion criteria consisted of age between 20 and 40 years, follicle stimulating hormone levels less than 10mLU/mL, body mass index (BMI) less than 35 kg/m^2 ^and suitable ovarian response during the IVF cycle (estradiol levels between 1000 pg/ml and 4000 pg/ml on the day of the injection of HCG – having 2 or 3 grade a or b embryos).

Exclusion criteria consisted of severe endometriosis, hydrosalpinx, anatomical anomalies of the uterus (momma, polyps), mullerian anomalies, endocrine disorders (thyroid disorders, poly cystic ovary syndrome (PCOS)), failed implantation on previous IVF- Intracytoplasmic sperm injection (ICSI) cycles, and sever disorders in the male partner. 

Infertile couples with male dysfunction (excluding azoospermia and sever seminal fluid abnormalities) and occluded salpinx without hydrosalpinx which had never undergone therapy cycles were included in the study. Sever seminal fluid dysfunctions was defined as less than one million sperms, normal morphology less than 4%, and sperm motility less than 10%. Induction of ovulation was done by gonadotropin-releasing hormone (GnRH) antagonist protocol, consisting of the following steps: first, on the second or third day of the cycle 150 to 300 units of recombinant FSH (gonaL-F, merk, serono) were injected, then by starting the sixth or seventh day GnRH injection (250 micrograms–cetrotide–merck, serono) was started and one to two Hp-HMG (monogamous – 75 to 150 units) were added to the therapy. With observation of at least 3 follicles bigger than 18 mm, hCG injection was carried out and after 34 to 36 hours’ ovarian puncture was done. The follicular fluid resulting from the 2 to 3 follicles other than the first one, which was not admixed with blood, nor underwent follicular washing, was extracted and the follicle was isolated, then in another test tube the fluid was centrifuged. Two cc of the centrifuged fluid was injected using intrauterine insemination catheters (IUI) into the uterine cavity after the follicles were punctured. The same was done for the control group, without the injection of the follicular fluid. The medical teams responsible for the implementation of the procedures were the same in both groups. Primary results were defined as chemical pregnancy and implantation rates. Chemical pregnancy was diagnosed by determining the levels of beta hCG levels 16 days after injection (a negative beta hCG sample was attained form each patient at day zero of the study), clinical pregnancy was defined by the observation of the gestational sac with an embryonic pole, and the observation of the fetal heart beat in ultrasound sonography, implantation rate was defined as the proportion of observed gestational sacs to transferred embryos. All sonographies were done by the same clinician and the same equipment. 

Statistical analysis was performed by SPSS software package version 16.0 for windows (SPSS Inc., Chicago, USA). 

**Table1:** Frequency of different etiological factors for infertility in study groups

	**Intervention Group** **(55 patients)**	**Control Group** **(55 patients)**
Fallopian tube obstruction	13 (23.6)	14 (25.5)
Male Dysfunction (moderately)	12 (21.8)	16 (29.1)
Ovulation Dysfunction	5 (9.1)	6 (10.9)
Endometriosis (Moderately)	3 (5.5)	5 (9.1)
Low Ovarian Reserve	4 (7.3)	4 (7.3)
Others	18 (32.7)	10 (18.2)

• Data is shown as Frequency (%)

• Other factors were consisted of hormonal imbalances, combination infertility and sperm allergy.

Quantitative data were presented as mean ± standard deviation (SD), while qualitative data were demonstrated as frequency and percent (%). for statistical analysis, After determining distribution of continuous variables by KlomogrovSimirnov test, Independent sample t-test was applied to compare two group's results. Also collected data were studied using descriptive statistical methods, the mean difference test for independent groups, Chi Square^2 ^test or Fisher’s exact test. P value less than 0.05 was statistically considered significant in all steps.

## Results

Of all the patients, 55 were appointed to the intervention group and 55 to the control group, and all 110 patients finished the study. The mean age in the intervention group was 33.64 ± 3.55 years (26 to 40) and 32.24 ± 4.61 in the control group (21 to 41), (p = 0.08). Mean duration of infertility was 7.19 ± 3.20 (1 to 13) years for the intervention group and 7.45 ± 3.93 years (2 to 20) for the control group, (p = 0.71). The etiological factors for infertility among the two groups are listed in table 1, the most important being fallopian tube obstruction (23.6% in intervention group and 25.5% in control group) and moderate male dysfunction (21.8% in intervention group and 29.1% in control group). Also, the comparative results of the study are shown in table 2, eliciting an implantation rate of 11.52 ± 2.57 (0-66.7) for the intervention group and 18.79 ± 3.72 (0-100) for the control group (p = 0.36), also chemical pregnancy was reported 34.5 % in the intervention group and 41.8 % in control group (p = 0.43) and clinical pregnancy rates were 30.9 % percent in intervention group compared to 38.2 % in the control group (p = 0.42), it can be seen that there were no significant differences between the two groups in clinical and chemical pregnancy rates nor implantation rates.

**Table 2:** Comparison Between Intervention and Control Groups After IVF

		**Intervention Group** **(55 patients)**	**Control Group** **(55 patients)**	**P**
FSH Level (mIU/mL)		8.38 ± 1.88 (3.13-10)	8.34 ± 2.15 (4.1-9.8)	0.92
Estradiol Level (pg/mL)		1065.58 ± 567.82 (11-2600)	1139.70 ± 649.71 (360-3100)	0.59
Attained Oocytes		9.35 ± 3.27 (4-16)	9 ± 3.77 (3-18)	0.61
Formed Embryos		6.67 ± 2.86 (3-13)	5.98 ± 2.55 (2-12)	0.18
Transferred Embryos	Total	2.91 ± 0.62 (2-4)	2.73 ± 0.53 (2-5)	0.76
	Grade A	2.6 ± 0.76 (2-4)	2.73 ± 0.53 (2-4)	0.31
	Grade B	0.4 ± 0.12 (0-4)	0.29 ± 0.11 (0-3)	0.5
Chemical Pregnancy		19 (34.5)	23 (41.8)	0.43
Clinical Pregnancy		17 (30.9)	21 (38.2)	0.42
Implantation		11.52 ± 2.57 (0-66.7)	18.79 ± 3.72 (0-100)	0.36

• Data is shown as Mean ± Standard Deviation (Min-Max) and Frequency (%)

• FSH stands as Follicle-stimulating hormone

## Discussion

During the present study, the effect of injection of follicular fluid into the endometrial cavity during follicular puncture, on implantation and pregnancy rates was investigated. The results showed that flushing the endometrial cavity neither had a significant effect on pregnancy nor on implantation rates. 

Assisted reproductive technology has developed dramatically during time but still failure rates are high. There are many reasons to this, but one important reason is failure of implantation of the embryo (9). Therefore, methods for increasing implantation have been suggested. 

Tehraninejad et al. demonstrated that by adding blastocyst culture supernatant to the endometrium, the probability of live births was increased and premature abortion was dramatically reduced, but neither there was significant change in pregnancy, nor in implantation rates which fallowed the results of the present study (10). 

Prapas et al., injected embryo culture supernatant to the endometrial cavity, before the embryo transfer on the 3^rd^ or 5^th^ day, and found that this did not positively nor adversely affect the implantation rates likewise in the present study (11).

Zhu et al. also demonstrated a positive effect on pregnancy and implantation rates by injection of culture fluid, but there was no significant increase in implantation and fertility rates, alike the present study (12). 

Berkkanoglu also showed that endometrial flushing had no effect on implantation rates, but also demonstrated that the effect of flushing was not dependent on the grade of the embryo and grade A and B embryos, made no difference accompanying flushing, which was in accordance to the present study (13).

Hashish et al. flushed the endometrium in patients undergoing ICSI, but similar to the present study found no significant change in results (5). 

Contrary to the present study, Goto et al. showed that injection of the follicular fluid into the endometrium, in women undergoing their first ART experience, made a significant difference (14, 15). Clinical pregnancy rates were up to 87% from 48% (p = 0.006) and implantation rates had nearly doubled (71.9% compared to 37.8%), in this randomized control trial. There were no significant differences between the intervention and the control group and the two were closely matched, but the number of patients undergoing the experiment were low compared to the previous studies mentioned above (23 women in the intervention group and 25 in the control group), and in those studies patients entering the study were all over 31 years old, while the current study had a greater age range (the inclusion criteria for the present study was for women over 21 years old). Also worthy of attention, patients entering Goto’s study, had a lower history of infertility compared to the present study (7.1 ± 3.6 – 6.1 ± 3.1 compared to 7.19 ± 3.2- 7.45 ± 3.93, respectively), all of the previous differences could have an effect on the results of the study and act as a confounding factor. 

The theoretical basis for flushing the endometrium with follicular fluid is that the fluid has an intensifying effect on the replication and decidualization of the endometrial cells (16), also the fluid is rich in cytokines such as endothelial growth factors and vascular endothelial growth factor, and Leukaemia inhibitory factor (17), a factor activating numerous signaling pathways, possibly having a key role in the process of implantation. Worthy of attention, Mehta et al. showed there was also a connection between the properties of the follicular fluid released during ovulation and the quality of the oocyte released (18). Similar results have also been reached by Feng et al. (19) and it had been shown that the follicular fluid had an immune modulating effect which could favor implantation (20). 

Despite all of this the present study and the majority of the studies conducted previously did not show any significant positive effect related to injection of follicular fluid on implantation or pregnancy rates, in women undergoing ART (in this study IVF).

This study's suggestion could be that in the future studies higher number of participants would better include in project, and the effect of different grade embryos should also be studied even further. Studies should also put more emphasis on the etiological factors for infertility and the probable difference in outcomes related to this. The present study was conducted in only one center, so the results are not generalizable to a wide array of patients. Addressing the study question in a multicenter study with more patients would be of merit.

## Conclusion

This study was conducted to examine the effects of injection of follicular fluid in to the uterine cavity in sub-fertile women undergoing IVF, on implantation and pregnancy rates, although certain previous studies had demonstrated that this procedure could have a beneficial effect on implantation rates and clinical pregnancy, the present study showed that a minor improvement was seen in the group undergoing the injection of follicular fluid, though the change compared to the control group was not significant.
